# Editorial

**Published:** 1900-01-15

**Authors:** 


### EDITORIAL.

### CHANGE OF MANAGEMENT.

After an editorial career of forty-four years, in connection with the DENTAL REGISTER, circumstances have so shaped that it seems best that a change be made. This involves the passage of the journal, so far as its ownership and editorial management is concerned, into other hands. In 1850 the ownership of the DENTAL REGISTER OF THE WEST, which had been held by the late Dr. James Taylor up to that time, passed by ownership into the possession of Drs. J. Taft and G. Watt, of Xenia, Ohio. This transfer was made in September of that year. The REGISTER was owned, edited and published jointly by these purchasers till 1873, when the senior editor purchased Dr. Watt's interest and was from that time on the sole owner and editor. In 1860 the REGISTER was changed from a quarterly to a monthly—the second monthly dental journal in the world. The REGISTER is the second oldest dental journal extant, and is the only one in the world that has had a continuous existence for so long a period. The REGISTER has never missed an issue of a single number from its beginning in 1847 to the present time. The *American Journal*, which is the oldest, had a lapse of seven years in its issue, namely, from 1860 to 1867.

In 1859 the REGISTER was transferred to Jno. T. Toland, of Cincinnati, who owned and published it. J. T. Toland was for several years successfully engaged in the dental supply business. In 1861 he entered the army, and there lost his life in a battle at Wytheville, W. Va., July 18, 1863. After this the proprietorship of the REGISTER again passed into the possession of the former owners, Taft and Watt. Soon after this the publication of the REGISTER was committed to the successors of Toland; the first of which was

J. S. Walters & Co., this firm was succeeded by Geo. Watt & N. W. Williams, and they by Spencer & Moore, and they by Spencer & Crocker; and following them was the present firm of Sam'l A. Crocker & Co., who have published the REGISTER for about fourteen years. The ownership of the journal has now passed into the possession of Dr. Theo. Menges, of the North-Western University Dental School. Its editorial management, is to be in charge of Dr. N. S. Hoff, of the Dental Department of the University of Michigan, and its publication will be in Cincinnati as heretofore, by Samuel A. Crocker & Co. It is certainly not without regret, loneliness, and a feeling of home-sickness that I vacate the editorial chair. In sundering this relation I shall greatly miss the opportunity and privilege of counseling with my professional brothers, from whom it has been my happy privilege to receive the kindest consideration, which has made smooth and pleasant the editorial pathway, and indeed given zest to my professional life that can never be forgotten. A relationship, that always has been attended with pleasure, and of more than forty years duration, is not broken without a tinge of regret. Most things in life have their compensations, however, and some are found in this case. It brings relief from labor and responsibility, and it gives opportunity for taking up work that has been already too long delayed. Another pleasant thought is, that the REGISTER will gain much by the change. Its life will be renewed. Those having it in charge will put into it the zeal and enthusiasm of a new enterprise. We sincerely hope that the DENTAL REGISTER may have in the future a bright and useful career—one commensurate with the growth and needs of the profession.

J. TAFT.

As will appear from the above editorial, Dr. Taft discontinues with this number his editorial connection with the REGISTER, and we assume the duties. We do so with no little mental and moral misgivings, because we doubt our ability to assume so exalted a station as the one just vacated by our illustrious and capable predecessor, and we doubt our capacity to patiently and resolutely meet all the vexatious problems that we understand are likely to meet one who assumes to cater to the capricious demands of an exacting and perhaps already to well supplied professional appetite. We shall not make any brave promises that we may in the future regret, and that may cause others to prognosticate our end. But we assume the duty as it has come to us, unsolicited on our part, and mean to be as faithful to the trust as our mental, moral and physical resources permit. We shall endeavor to do our work well, and in accord with what we believe to be the higher ideals of the profession. Whether our career shall be a long or a brief one, we shall always be influenced in sentiment if not in action by those who have so ably conducted the editorial pages of this journal before us. We do not expect to approach the devotion of Dr. Taylor, or wield the strong and witty pen of Dr. Watt, nor to possess the broad and comprehensive grasp of professional affairs that characterized the work of Dr. Taft. But we shall be content to serve the profession to the best of our ability, using this instrument providentally placed in our hands, with the help promised us from those more able than we are, to level up the general standard of professional intelligence. We trust we shall ever maintain the high professional ideals which have characterized the DENTAL REGISTER in all its past history.

---

BY way of apology for the tardy appearance of the REGISTER this month, we can only say that the matters of transfer of property and arrangements for publication and editorship were completed several days since the beginning of the year, and consequently the time has not been sufficient for a systematic arrangement of the matter as we design to present it in the future.

---

The seventh annual meeting of the National School of Dental Technics was held in Philadelphia, December 27th, 28th and 29th. In many respects it was the most interesting meeting this organization ever held. The question as to its sphere of action was we think settled, so that in future it will be recognized as the organization of all others best qualified to consider educational methods and principles. Hereafter it will be expected of this organization that all dental pedagogical matters can be discussed in its meetings without fear of encroaching on the sphere of any other organization. The meeting was representative, as twenty-two schools were represented. The University of California sent three delegates. As indicating a tendency to broaden the scope of the organization and make it more specific and comprehensive, the name was changed to *The Institute of Dental Pedagogy*. Another important action was the decision to make a display at the Paris Exposition, which shall represent the curriculum and teaching facilities of the American Dental Colleges. This exhibit will be a composite and not an individual display. Every paper scheduled by the printed program, with but a single exception, was presented, and this exception was the result of a misunderstanding. It is the intention to hold the next meeting at Nashville and the Executive Board has already begun to outline the program. Philadelphia as a meeting place was all that was expected, and the faculties of the several schools were untiring in their efforts to afford us every opportunity to prosecute the business of the body as well as to partake of their hospitality. The opportunity to visit and inspect the buildings and facilities of the several schools was well worth the trouble and cost of the trip.

